# Airway inflammation after inhalation of nano-sized zinc oxide particles in human volunteers

**DOI:** 10.1186/s12890-019-1026-0

**Published:** 2019-12-30

**Authors:** Christian Monsé, Monika Raulf, Olaf Hagemeyer, Vera van Kampen, Benjamin Kendzia, Vitali Gering, Eike-Maximilian Marek, Birger Jettkant, Jürgen Bünger, Rolf Merget, Thomas Brüning

**Affiliations:** Institute for Prevention and Occupational Medicine of the German Social Accident Insurance, Institute of the Ruhr University Bochum (IPA), Bürkle-de-la-Camp-Platz 1, 44789 Bochum, Germany

**Keywords:** Zinc oxide, Nanoparticles, Induced sputum, Local effects, Inhalation study

## Abstract

**Background:**

Workers in the zinc production and processing of galvanized sheet steel are exposed to a complex mixture of particles and gases, including zinc oxide (ZnO) that can affect human health. We aimed to study the effects of short-term controlled exposure to nano-sized ZnO on airway inflammatory markers in healthy volunteers.

**Methods:**

Sixteen subjects (8 females, 8 men; age 19–42, non-smokers) were exposed to filtered air and ZnO nanoparticles (0.5, 1.0 and 2.0 mg/m^3^) for 4 h, including 2 h of cycling with a low workload. Induced sputum samples were collected during a medical baseline and a final examination and also about 24 h after each exposure. A number of inflammatory cellular and soluble markers were analyzed.

**Results:**

Frequency and intensity of symptoms of airway irritation (throat irritation and cough) were increased in some subjects 24 h after ZnO exposures when compared to filtered air. The group comparison between filtered air and ZnO exposures showed statistically significant increases of neutrophils and interleukin-8 (IL-8), interleukin-6 (IL-6), matrix metalloproteinase (MMP-9) and tissue inhibitors of metalloproteinases (TIMP-1) in sputum starting at the lowest ZnO concentration of 0.5 mg/m^3^. However, a concentration-response relationship was absent. Effects were reversible. Strong correlations were found between neutrophil numbers and concentrations of total protein, IL-8, MMP-9, and TIMP-1.

**Conclusions:**

Controlled exposures of healthy subjects to ZnO nanoparticles induce reversible airway inflammation which was observed at a concentration of 0.5 mg/m^3^ and higher. The lack of a concentration-response relationship warrants further studies.

## Background

Inhalation of zinc oxide containing atmospheres (for example welding fumes) may induce zinc fever. Besides fever, typical symptoms include throat irritation, cough, minor respiratory symptoms, metallic taste, as well as flu-like symptoms [1]. Typically, the symptoms occur after a latency period of 4–12 h, and resolve within 48 h. While a number of older studies reported medical effects after very high exposures to ZnO [2, 3], no effects were detectable at 0.5 mg/m^3^ ZnO [4]. However, with concentrations between 1.1 and 1.5 mg/m^3^ ZnO-containing welding fumes significant increases of C-reactive protein (CRP) in blood, a marker of inflammatory processes, were reported [5]. A recent study also showed increases in inflammatory mediators in the nasal mucosal lining fluid with the aforementioned concentrations [6]. Krabbe et al. [7] conducted repetitive exposures to welding fumes and demonstrated a persistent increase of systemic inflammatory markers that indicates an elevated risk for workers chronically exposed to zinc- and copper-containing welding fumes.

The first manuscript of this study [8] demonstrated a concentration-response relationship of zinc oxide (ZnO) nanoparticles concerning systemic effects. Whereas no relevant effects were detectable after sham exposures and 0.5 mg/m^3^ ZnO, reversible effects on acute phase proteins and neutrophils in blood occurred after 1.0 and 2.0 mg/m^3^ ZnO exposure. Effects were strongest after 2.0 mg/m^3^ ZnO, with flu-like symptoms and elevated body temperature in several subjects. ZnO exposure showed no detectable effects on fractional exhaled nitric monoxide (FeNO) and lung function parameters. In summary, the effects measured in the first part of this study were indicative of reversible acute systemic inflammation caused by a zinc ion-specific mechanism [9].

As the first contact of ZnO after inhalation occurs in the respiratory tract, it is plausible to investigate local (airway) effects in addition to systemic effects. These effects were determined simultaneously with the systemic effects in the same subjects and same exposure conditions as described previously [8]. We decided to perform sputum induction by inhalation of isotonic saline as a non-invasive, simple and safe procedure to collect material from the lower airways following a consensus statement that was reached by a panel of experts on non-invasive methods for assessment of airway inflammation in the studies of occupational respiratory diseases [10]. Some studies have shown that induced sputum is a useful diagnostic tool to detect local effects such as increased neutrophil numbers and concentrations of soluble inflammatory markers after inhalation of harmful substances, not only in experimental human studies, but also in field studies of e.g. bitumen-exposed workers [11]. To our knowledge there is only one experimental human exposure study with ZnO inhalation in a low concentration range (0.5 mg/m^3^ for two hours at rest [4]) with included sputum analysis. That study did not show any inflammatory effects. In addition, only few inhalation studies with materials such as carbon black, diesel motor emissions, concentrated ambient particles or alumina including sputum analyses were published (see Discussion). None of these studies used more than one concentration step, with the exception of one study with a negative outcome [12] and thus, information about concentration-response relationships in sputum samples is not available.

It is known that neutrophilic inflammation is a major characteristic of metal fume fever [13]. Thus, this study focused on biomarkers which are associated with neutrophilic inflammation. It was the aim of this study to assess if neutrophilic inflammation can be induced by mono-exposure to nano-sized ZnO particles and to describe the concentration-response relationship in a multiple steps design in a low exposure range.

## Materials and methods

### Study design and participants

Potential volunteers were tested for their suitability to participate in the study in a baseline examination including a questionnaire, medical examination, lung function test, exercise testing as well as collection of blood and induced sputum. All sera were tested for specific IgE antibodies (sIgE) to ubiquitous allergens using ImmunoCAP (sx1, Thermo Fisher Scientific, Uppsala, Sweden). Specific sIgE concentration of ≥0.35 kU/L was considered positive.

The study participants had to be able to produce sputum according to our criteria (eosinophils < 1%, epithelial cells < 95% and neutrophils not dominant) in order to exclude asthmatics and to make sure that the material originated from the lower airways. They should be at best not sensitized to ubiquitous allergens (sx1 negative). However, because healthy and non-smoking subjects can produce relatively poor amounts of induced sputum, we had to examine more than 60 subjects to find 16 suitable ones. Out of them 7 were sx1 positive (median 3.64 kU/L (range: 0.96–16.2 kU/L)) and all were sensitized to seasonal (pollen) but not to perennial allergens. Thus, the study was performed out of pollen season.

In total sixteen healthy non-smoking subjects were exposed for 4 h to ZnO particles with different concentrations (sham, 0.5, 1.0 and 2.0 mg/m^3^ ZnO). Details of the ZnO particle generation were already described in [14], the particle sizes were comparable with those of an emission study of galvanized materials with different welding techniques [15], and investigations on the homogeneity of the ZnO atmospheres were presented in [16]. Sham exposures (0 mg/m^3^ ZnO) were also performed with the in [14] described flame generator operating with purified water without zinc salt. This procedure ensured that the same concentrations of nitrogen oxides (NO_x_) were present at all exposure levels which were generated as trace gases by the pyrolysis process. Other measured trace gases are not expected to confound the medical effect parameters in human exposure studies [14].

Further specific details of the study design, including the characterization of the subjects and the activity on cycle ergometers were described elsewhere [8].

### Questionnaire

All subjects answered a questionnaire addressing a variety of symptoms. In this study only symptoms indicating airway irritation (throat irritation and/or cough) are reported. Both symptoms were graded according to severity (not at all (0 score point), barely (1 points), little (2 points), moderate (3 points), strong (4 points), very strong (5 points)).

### Induced sputum

Sputum samples were obtained at the baseline examination, 24 h post-exposure and at the final examination, but not directly before exposures. This procedure eliminates the possibility that repeated sputum recovery within a short time period may induce inflammatory effects triggered by sputum induction itself. According to the procedure used in several studies [10, 17, 18] sputum induction was carried out by 15 min inhalation of nebulized isotonic saline solution (0.9% sodium chloride (NaCl)) (Pariboy, Pari GmbH, Weilheim, Germany). Afterwards the subjects tried to cough up sputum. For the processing of the sputum samples, the whole sputum technique was used. The collected sputum material was processed within 2 h after sampling, weighed, mixed with 2.5-fold quantity of 0.1% sputulysin solution (6.5 mM dithiothreitol solution in 100 mM phosphate buffer, Calbiochem GmbH, Bad Soden, Germany) to allow mucolysis and disperse the sputum cells, slowly vortex the sample for 30 s and incubated in a water bath at 37 °C for 30 min. After centrifugation (400 x g, 10 min, 4 °C) the cell-free supernatant was aliquoted and frozen at − 80 °C until further analysis of the soluble biomarkers. The remaining cell pellet was re-suspended, and the cell number was quantified using the Neubauer counting chamber. Cytospin preparations were prepared for differential cytology and stained with May-Grünwald-Giemsa solutions. 300 sputum cells were microscopically differentiated by two independent evaluators each at a magnification of 1:100 under oil immersion. Results were expressed as percentage for each cell population (macrophages, neutrophils, eosinophils, basophils, lymphocytes and epithelial cells) and absolute numbers calculated from the total cell count. The quality of the cytospin sputum preparations was controlled with regard to contamination by squamous cells (mostly coming from the mouth). If the percentage of squamous cells was greater than 80%, the sample was not taken into account. Based on the previously defined exclusion criteria for the study participants, the percentage of eosinophils in 96% of the tested samples was below 1%. Basophils could not be detected in any sputum sample. For the evaluation of cellular changes we focused mainly on neutrophils.

Inflammatory markers were quantified in the thawed cell-free supernatants of the aliquots. All samples underwent only a single freeze-thaw cycle. Concentrations of IL-6, IL-8, MMP-9, TIMP-1, 8-iso-PGF_2α_ and Substance P were determined in the appropriate immunoassays based on monoclonal or polyclonal antibodies (Pharmingen, Heidelberg, Germany, Assay Design and/or Bio Vendor, all: Heidelberg, Germany) according to the recommendations of the manufacturers. The total protein determination was carried out with bovine serum albumin as a standard with a measuring range of 10 to 100 mg/L [19]. All samples were measured in up to three different dilutions and results were accepted if the coefficient of variation (CV) was below 25%, otherwise they were repeated. The respective lower quantification limit was 3 pg/mL for IL-8, 4.7 pg/mL for IL-6, 31.2 pg/mL for MMP-9 and TIMP-1, 9.76 pg/mL and 6.1 pg/mL for Substance P and 8-iso-PGF_2α_.

### Data analysis

Descriptive analysis was performed for each variable stratified by exposure (sham, 0.5, 1.0 and 2.0 mg/m^3^ ZnO) and time of measurement (baseline examination, final examination, and 24 h post exposure). Characteristics of subjects were expressed as medians, 25%- and 75%-quantiles, as well as minimum and maximum. Graphical representations were illustrated with boxplots. Sputum parameters were compared between 24 h after sham exposure and 24 h after ZnO exposures. To estimate the effects of ZnO on the sputum parameters we used various generalized estimating equations (GEE) models, but the algorithm did not converge (data not shown). Therefore exposure groups (sham / ZnO) comparisons were performed with paired Student’s t-test for normal distributed variables. In case of skewed data the paired Student’s t-test was used after log-transformation. The problem of multiple comparisons was counteracted using the Bonferroni correction [20], by dividing the overall desired statistical significance level α = 0.05 by the number of hypotheses tested.

Spearman correlation coefficients (r_S_) with 95% confidence intervals (95% CI) were calculated to predict the monotone association between parameters. *P* values were calculated using the specified null hypothesis r_S_ = 0.

Rank order tables were developed to give another estimate of increased effects which follow a concentration-response relationship. Increased effects were defined as values bigger than the largest value of baseline examination, final examination and sham exposure (*n* = 3) plus the double median absolute deviation (MAD) of these 3 values (> max (baseline examination, final examination, 24 h after sham exposure) + 2 MAD). Each of the ZnO related effects was assigned to ranks 1 to 4, the lowest value represented by rank 1 and the highest by rank 4, respectively. All parameters of induced sputum were evaluated with the group comparison and rank order tables.

## Results

### Questionnaire

There was a tendency for symptoms to increase after all ZnO exposures, with the greatest increase after the highest exposure concentration. A concentration-response relationship was absent (Table 1).

**Table 1:** Symptoms of airway irritation reported by questionnaire (at least 1 of 2 symptoms: throat irritation and cough) according to ZnO concentrations and time points. Both symptoms were graded according to severity (not at all (0 score point, not illustrated), barely (1 points), little (2 points), moderate (3 points), strong (4 points), very strong (5 points)). Listed values are sums of both graded symptoms (the maximal value is 10)

ZnO-Conc. [mg/m^3^]		0			0.5			1.0			2.0	
Time points	**A**	**B**	**C**	**A**	**B**	**C**	**A**	**B**	**C**	**A**	**B**	**C**
ID												
1				–	1							2
2	1			2			2	1	4	1		
3			1	2	1		1	2	1		4	4
4												
5				2	2	1		1	1	2	1	
6		1			1	2		1		1	3	2
7					1	4	1	–	3		1	4
8												
9	1				2	4			2			
10					1				1			
11				1		1						2
12												
13		1				2			4	1	2	3
14												
15							2	1				
16	1	1	1	1			1	1				3
Sum score	3	3	2	8	9	14	7	7	16	5	11	20
% of max. Sum score (160)	1,9	1,9	1,3	5,0	5,6	8,8	4,4	4,4	10,0	3,1	6,9	12,5

Time points A: before exposure, B: directly after exposure, C: 24 h after beginning of exposure. Hyphens indicate missing values. The maximum value of 160 would be reached if both symptoms are reported by all subjects with highest severity

### Induced sputum

The results obtained from baseline examinations, 24 h after sham, and from final examinations were not significantly different from each other (data not shown). In addition, the results of the final examinations that were conducted at the end of the study (minimum 14 days after the last exposure) showed that all parameters had returned to levels within the range of the baseline values.

**Fig. 1:**
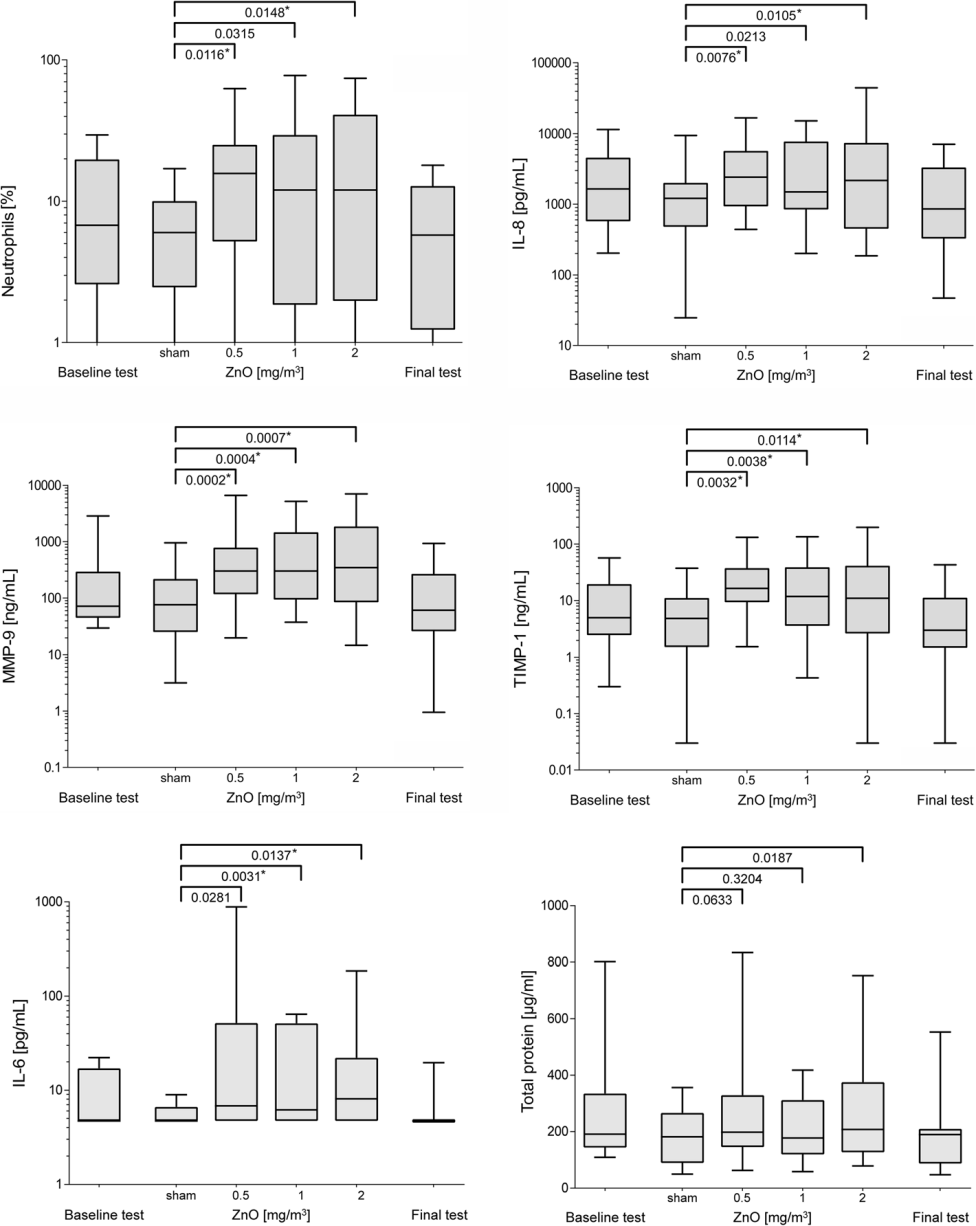
Boxplots of sputum parameters with at least one significant increase (*p* < 0.05). Fifty out of 92 measurements were below LOQ for IL-6. Significance level after Bonferroni correction is α = 0.0167. Asterisked *p*-values are statistically significant

Only those parameters with at least one increase with a significance level *p* < 0.05 are presented in Fig. 1. ZnO exposure had no significant effect on concentrations of 8-iso-PGF_2α_ and Substance P in induced sputum after 24 h of exposures.

Statistically significant increases of neutrophils and IL-8 concentrations after Bonferroni correction were observed by comparing sham with 0.5 mg/m^3^ ZnO and sham with 2.0 mg/m^3^ ZnO, but not when comparing sham with 1.0 mg/m^3^ ZnO (Fig. 1). Absolute and relative numbers of neutrophils yielded similar results (absolute numbers not shown). Compared to sham exposure, the increases of MMP-9 and TIMP-1 concentrations were seen for all exposures to ZnO (also after Bonferroni correction). The increase in total protein concentration was not statistically significant 24 h after exposure between sham and 0.5 mg/m^3^ ZnO and sham and 1.0 mg/m^3^ ZnO, but the comparison between sham exposure and 2.0 mg/m^3^ ZnO was almost statistically significant (*p* = 0.0187). Statistically significant increases (after Bonferroni correction) of IL-6 concentrations were observed by comparing sham with 1.0 mg/m^3^ ZnO and sham with 2.0 mg/m^3^ ZnO, but not by comparing sham with 0.5 mg/m^3^ ZnO. For all parameters a concentration-response relationship could not be demonstrated: neutrophils, IL-8, IL-6, MMP-9, TIMP-1 and total protein did not show significant differences by comparing the ZnO exposure concentrations among themselves (0.5 vs 1.0, 0.5 vs 2.0, 1.0 vs 2.0 mg/m^3^ ZnO, data not shown).

Correlations between sputum parameters are listed in Table 2. Spearman correlation coefficients (r_S_) represent the results of the analyses using the averaged values of sham and all ZnO exposure concentrations. The correlations within a single ZnO concentration were similar (data not shown). It is striking that almost all parameters were correlated with each other (maximum r_S_ was 0.95). Substance P was negatively correlated with almost all other parameters.

**Table 2:** Spearman correlation coefficients (r_S_) between total cell number, percentage of neutrophils, total protein and inflammatory markers in induced sputum (values 24 h after all ZnO exposures were considered) with 95% confidence intervals and p-values

	Total cell number	Neutrophils [%]	Total Protein [μg/mL]	IL-8 [pg/mL]	MMP-9 [ng/mL]	TIMP-1 [ng/mL]	8-iso-PGF_2α_ [pg/mL]	Substance P [pg/mL]	IL-6 [pg/mL]
Total cell number [× 10^5^]	1	0.51* 0.35–0.67 < 0.0001	0.52* 0.37–0.67 < 0.0001	0.44* 0.28–0.61 < 0.0001	0.60* 0.46–0.73 < 0.0001	0.58* 0.45–0.71 < 0.0001	−0.07 -0.26-0.13 0.4931	−0.21* -0.40- -0.02 0.0315	0.35* 0.18–0.52 0.0002
Neutrophils [%]		1	0.70* 0.60–0.78 < 0.0001	0.69* 0.58–0.82 < 0.0001	0.81* 0.73–0.89 < 0.0001	0.79* 0.70–0.87 < 0.0001	0.17 -0.02-0.36 0.3120	−0.26* -0.46- -0.07 0.0008	0.61* 0.48–0.75 < 0.0001
Total protein [μg/mL]			1	0.85* 0.78–0.92 < 0.0001	0.84* 0.73–0.89 < 0.0001	0.88* 0.82–0.96 < 0.0001	0.34* 0.19–0.50 0.0003	−0.23* -0.42- -0.04 0.0193	0.56* 0.43–0.70 < 0.0001
IL-8 [pg/mL]				1	0.87* 0.82–0.95 < 0.0001	0.90* 0.85–0.96 < 0.0001	0.37* 0.22–0.52 0.0001	−0.29* -0.48- - 0.10 0.0046	0.59* 0.47–0.71 < 0.0001
MMP-9 [ng/mL]					1	0.95* 0.94–0.97 < 0.0001	0.24* 0.08–0.40 0.0146	−0.28* − 0.46- -0.09 0.0046	0.68* 0.58–0.77 < 0.0001
TIMP-1 [ng/mL]						1	0.19* 0.02–0.35 0.0497	−0.33* -0.41- -0.15 0.0008	0.65* 0.54–0.77 < 0.0001
8-iso-PGF_2α_ [pg/mL]							1	0.17 -0.03-0.38 0.0791	0.05 -0.14-0.23 0.6389
Substance P [pg/mL]								1	−0.24* -0.44- -0.03 0.0146
IL-6 [pg/mL]									1

Correlations with *p* < 0.05 are marked with asterisks

As shown by rank order tables increases of parameter concentrations in induced sputum were found for all ZnO concentrations in almost all subjects (gray colored cells in Table 3). A concentration-response relationship was absent in most subjects on an individual basis (indicated by the numbers 1–4 in Table 3) and could be found only in three subjects for neutrophils, two for IL-8, 4 for MMP-9, one for TIMP-1, and one for IL-6. These relationships were independent of subject’s ID, except for ID 9, who showed concentration-response relationships in three parameters (neutrophils, IL-8 and MMP-9). Increased effects were detected in nine of 96 measurements (9.4%) after sham exposure for three subjects for IL-8, three subjects for MMP-9 and three subjects for TIMP-1.

**Table 3:**
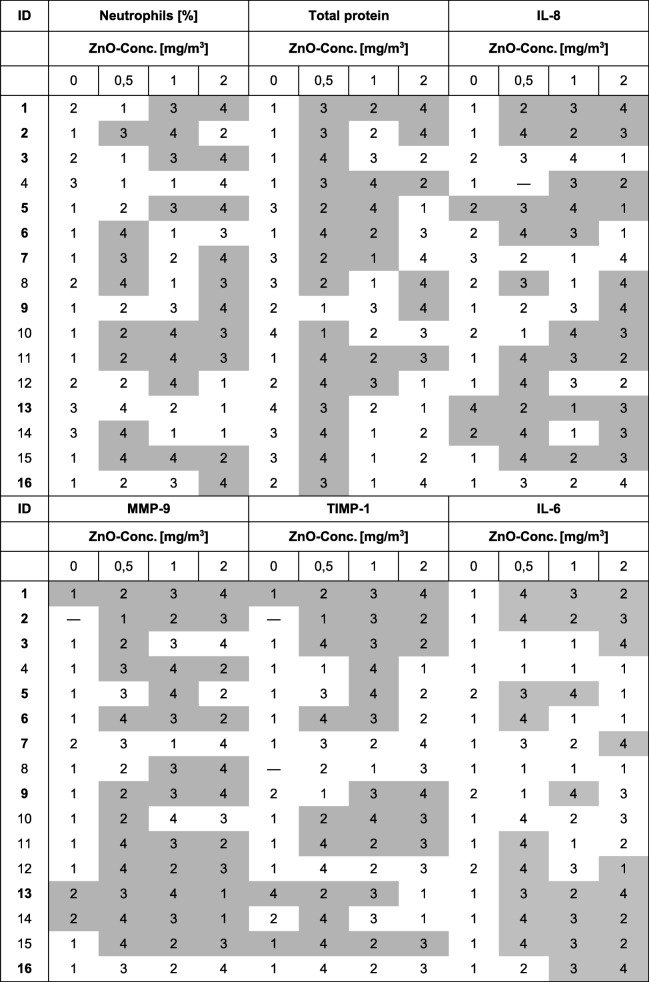
Rank order tables

Grey colored boxes represent increased effects defined as > max value of baseline examination, final examination and 24 h after sham plus double median absolute deviation (MAD) of these 3 values. Numbers indicate ranks of effects. Hyphens indicates missing values. Bold IDs are subjects who reported symptoms in the questionnaires

**Fig. 2:**
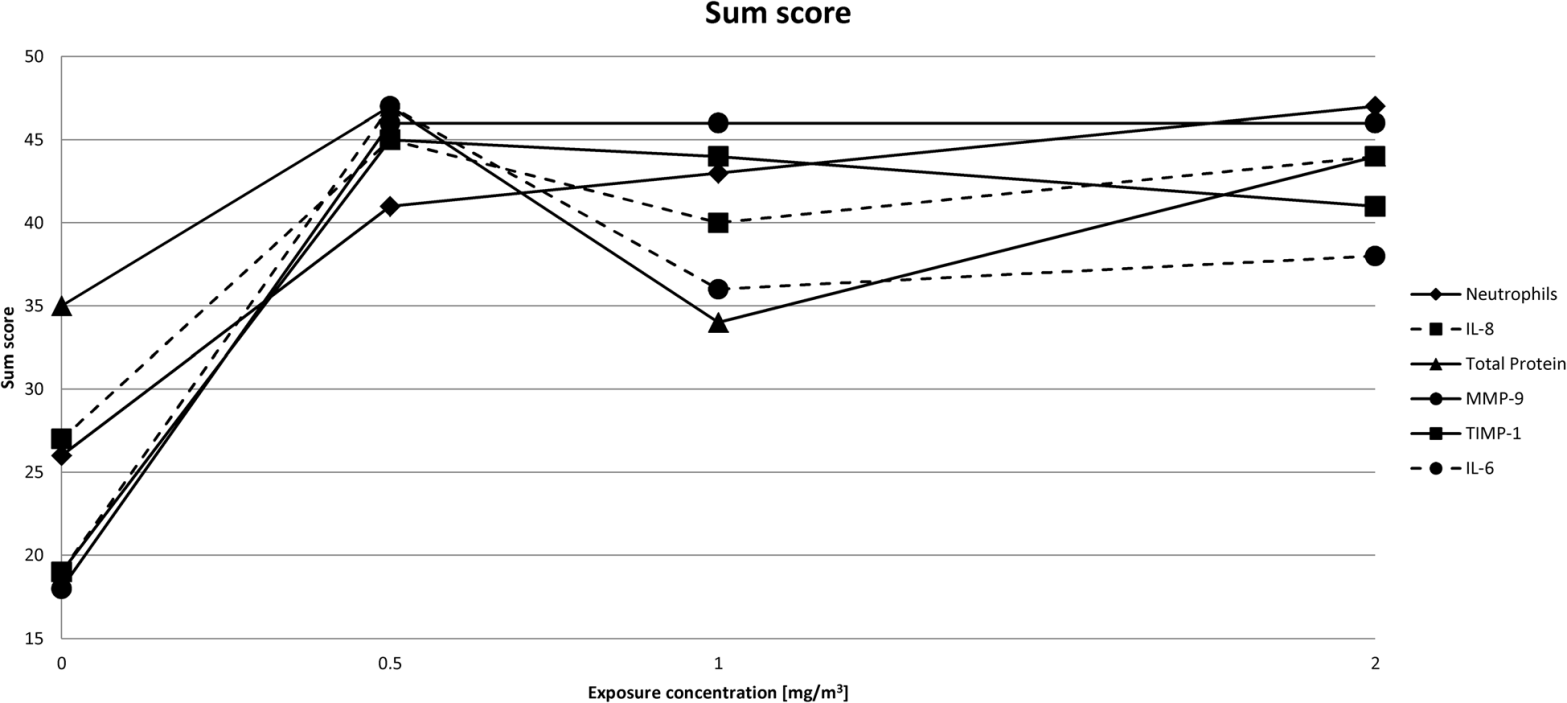
Sum score of ranks of selected sputum parameters. Sum scores were generated by addition of the rank values of the respective ZnO concentrations in Table 3

Finally, sum scores were generated by addition of the rank values of the respective ZnO concentrations in Table 3 and were graphically displayed (Fig. 2). The height of the sum scores does not reflect the quantitative concentrations of the selected sputum parameters, but merely serves to qualitatively classify the effects. Also the sum scores of the ranks showed no concentration-response relationship.

There were no differences between males and females, as well as between subjects with and without sensitizations to ubiquitous environmental allergens (data not shown).

## Discussion

Experimental inhalation studies in humans with nanoparticles that use multiple concentration steps in order to describe a concentration-response relationship are extremely sparse. The present study is, to our knowledge, the first human inhalation study that examined beside systemic effects [8] several parameters in induced sputum in a multi-concentration design of 0, 0.5, 1.0 and 2.0 mg/m^3^ ZnO. The key result of this study is the demonstration of an inflammatory airway response without a concentration-response relationship at a concentration of 0.5 mg/m^3^ and higher. Effects were seen for some parameters (neutrophils, IL-8, IL-6, MMP-9 and TIMP-1) in induced sputum. All of them were reversible as demonstrated at the final examination about two weeks after the last ZnO exposure. Few effects were detectable after sham exposures due to high spontaneous variability (see Table 3). The sputum parameters neutrophils, total protein, IL-8, MMP-9, and TIMP-1 were highly correlated. 8-iso-PGF2α, Substance P, and IL-6 showed weak but significant correlations. The first part of this study [8] had shown that FeNO and lung function parameters were not affected by ZnO exposures.

Symptoms of airway irritation were used as a secondary outcome. As a tendency, mild symptoms of airway irritation such as throat irritation and cough were reported by a higher number of subjects 24 h after ZnO exposures with the greatest increase after the highest exposure concentration. However, even the highest percentage sum score value of 12.5% is relatively low, although the maximal averaged sum scores in this study were about double of the averaged sum scores of time points without any previous ZnO exposures. In order to quantify this sum score, a method for the assessment of sensory irritation was chosen: the Scientific Committee on Occupational Exposure Limits [21] used a five step questionnaire: (1) no effects observed, no awareness of exposure; (2) very slight effects, awareness of exposure; (3) slight irritant effects or nuisance (e.g. odour), easily tolerable; (4) significant irritation/nuisance, overt health effects, barely tolerable; (5) serious health effects, intolerable. SCOEL interprets an effect as adverse being between step 2 and 3. Transferred to this study, a percentage averaged sum score value between 40 and 60% would be considered adverse, but few subjects reported symptoms corresponding to SCOEL grades 2 or 3 symptoms.

In addition, the responses of some subjects were not consistent, as symptoms were reported at lower exposure levels, but not at the highest ZnO concentration (ID 2, 5, 9, 10). Four out of 16 subjects (25%) did not indicate symptoms at any time point, and a considerable number of subjects (10 out of 16) reported symptoms without any previous ZnO exposures (after sham or before ZnO exposures). Overall, increases of symptoms after ZnO exposures in this study were considered minor.

Human experimental inhalation studies that used effect parameters in induced sputum showed considerable heterogeneity with respect to methodological issues like the inhaled substances, airborne concentrations, time points of sputum recovery, health status of the subjects as well as the effect parameters. Most of the studies covered diesel motor emissions, ambient particles in different experimental setups (untreated particles [22–25]) with or without the presence of nitrogen dioxide [26] or heated and non-heated collected particles [26]). Few studies used substances like ultrafine carbon particles [12], ZnO [4] or aluminium oxide (Al_2_O_3_) [27]. With the exception of the study by Sikkeland et al. [27] who conducted their experiments with a relatively high exposure concentration of 4 mg/m^3^ Al_2_O_3_, concentration levels in the studies ranged between 10 and 500 μg/m^3^. With one exception [12] only one single particle concentration was used in addition to the sham exposure. In that study experiments were performed in different groups with ultrafine carbon particles in a complex study design. In most studies sputum was obtained approximately 24 h (range 20 to 30 h) after exposure. Information was given in almost all studies on the total and differential cell count. In a large part of the studies, various markers like cytokines were determined. Often increases of neutrophils and several soluble mediators (e.g. IL-6, myeloperoxidase, methylhistamine) were reported in healthy humans. These effects were independent of the substances. In the only available ZnO study [4] in which sputum analyses were carried out, neither systemic nor airway effects were observed after inhalation of 0.5 mg/m^3^ ZnO for two hours without physical exercise, resulting in an approximately 4 fold lower inhaled dose (with linear extrapolation) of ZnO in comparison with the lowest ZnO dose in this study.

Pietropaoli et al. [12] examined three different ultrafine carbon particle concentrations (in different groups) but they were not able to demonstrate any effects on the measured parameters. However, the concentrations were very low (10, 25, and 50 μg/m^3^). Sikkeland et al. [27] were the only working group reporting the measurement of the inflammatory parameter MMP-8, but found no change after exposure although a very high concentration of Al_2_O_3_ (4 mg/m^3^) was used. Thus, the results of these studies do not allow to answer the question whether concentration-response relationships exist for sputum parameters in general and ZnO in particular.

Reference values for sputum parameters do not exist and concentrations show large inter-individual variability. Thus, descriptive analyses with respect to reference values were not considered in this study. Instead, group comparisons (sham / ZnO) and additional intra-individual analyses by rank order tables were used. The latter were developed in order to overcome the problem of multiple testing and associated definitions of significant effects. For all sputum parameters three control scenarios were available (baseline examination, 24 h after sham exposure, final examination), thus accidental variabilities were minimized in this study

One possible weakness of the present study is that the effect parameters were recorded at limited time points. It can therefore not be excluded that some effects might be overlooked due to their shorter or longer kinetics. However, the time points of the increases in the most sensitive parameters - neutrophils, IL-8, MMP-9, and TIMP-1- in this study correspond well with other evaluations of neutrophils [25, 27–31]. A further aspect is the lack of blinding of the exposure with the highest ZnO concentration [8]. In general, inhalation studies should be blinded, but for security reasons due the high ZnO concentrations, this was not performed. However, confounding of objective parameters in induced sputum should be negligible.

Sixty persons were screened before 16 suitable subjects were found who were able to produce sputum and who should be at best not sensitized to ubiquitous allergens according to the present criteria. This may represent a selection, but our focus was on a complete data set. It is not known that effect differences exist between healthy subjects that can deliver sputum and those who cannot.

The strength of this study is the low number of effects after sham exposures (0 mg/m^3^ ZnO) with increased effects in only nine of 96 measurements (9.4%). In contrast, 58.3% (0.5 mg/m^3^ ZnO), 57.3% (1.0 mg/m^3^ ZnO), and 56.3% (2.0 mg/m^3^ ZnO) of the measurements showed increased effects. The highly correlated sputum parameters neutrophils, total protein, IL-8, MMP-9, and TIMP-1 add to the credibility of our study results. Many control conditions were performed without ZnO exposure (baseline examination, sham exposures and final examination). This has the consequence, that the intra-individual variations of the parameters can be determined that play a role in the statistical evaluation and that the influence of released trace gases (NO_x_) during ZnO nanoparticle generation on medical parameters can be excluded, since there are no statistical differences between the analyses of the initial/final examinations and after sham exposures. The number of 16 subjects was sufficient, as they were exposed several times to ZnO and sham, thus serving as their own control. Since the results presented here show statically significant changes, we have refrained from presenting the power calculation we carried out in advance.

Based on our experience with sputum collection in several field and controlled exposure studies [11, 17, 18, 31–33] we used inhalation of 0.9% saline for 15 min for sputum induction. In our view this is a suitable non-invasive and non-stressing procedure to collect material from the airways with satisfying recovery and typical composition of cellular and soluble components in most of the healthy subjects. In addition to the determination of the cellular profile of the sputum samples we determined several established soluble biomarkers characterizing inflammation (IL-8, IL-6, LTB_4_), oxidative stress (e.g. 8-iso-PGF_2α_) and structural changes that contribute to bronchial mucosa remodeling, such as MMP-9 and TIMP-1. Several of these biomarkers are involved in the inflammatory process in obstructive lung disease and their concentrations are increased also in sputum samples of healthy smokers compared to nonsmokers [11]. As other studies we included the tachykinin Substance P as soluble sputum parameter, a neuropeptide playing an important role in the development of airway inflammation upon both allergen challenge and inhalation of irritants in animal models [34]. In the majority of the induced sputum samples (77.5%) the concentration of Substance P was above the detection limit with a high degree of individual variability without significant ZnO exposure effect.

In summary, this manuscript reports reversible concentration-independent changes of parameters for inflammatory processes in the airways in healthy subjects after inhalation of nano-sized ZnO particles at the lowest concentration of 0.5 mg/m^3^, a concentration which did not induce metal fume fever or significant inflammatory systemic effects in the same subjects [8].

As there are no comparative data from other studies on the importance of changes in the concentration of sputum parameters with stepwise increasing particle concentrations, it remains to be confirmed by further studies whether increases of inflammatory markers without a concentration-response relationship can be reproduced. Epidemiologic studies of subjects with exposure to ZnO are inconclusive due to co-exposures to further irritants e.g. in welders or electroplaters.

Increased levels of markers of airway inflammation (e.g., polymorphic neutrophils or inflammatory cytokines in bronchoalveolar lavage or sputum) were regarded as potentially adverse respiratory health effects of air pollution by an international expert group [35]. Very recently, increased pro-inflammatory mediators and recruitment/infiltration of leukocytes were defined as key events for use in adverse outcome pathways [36]. Such effects were found in this study. However, a concentration-response relationship, which is one criterion of causation of a disease by environmental exposures [37] was absent. The lack of a concentration-response relationship of the airway effects in the present study is difficult to explain. It cannot be ruled out that a concentration-response relationship might have been reached when different sampling periods were chosen. Since the initial contact with ZnO had taken place in the airways (portal of entry), earlier begin of local reactions should be expected compared to the systemic reactions. For practical reasons, we decided to collect both blood and sputum samples about 24 h after exposure. While we might have met the kinetics of blood parameters near their maxima [8], the maxima of the kinetics of the sputum parameters may have already been exceeded. Also, if the dose-response curve shows a plateau of the effect sizes, the exposure concentrations in this study might be already in the plateau part. To our knowledge there are no human exposure studies which support these hypotheses. Other explanations are possible, e. g. different kinetics or quantitative aspects. While the systemic effects are probably caused by zinc ions [9], it can be assumed that the local effects are triggered by both zinc ions and particles [38]. Based on the data obtained in this study and the data known in the literature, we are currently not in a position to identify a particular mechanism and these open questions need further study.

## Conclusion

Whereas clear concentration dependent systemic effects were detected after ZnO inhalation at and above 1 mg/m^3^ ZnO, increases of some airway inflammatory markers were measured at and above 0.5 mg/m^3^ in the same subjects, but a concentration-response relationship was absent. These results are strengthened by increases of several parameters indicating airway inflammation and high correlations between multiple cellular and soluble inflammatory markers. According to these results a No Observed Effect Level (NOEL) for nanosized ZnO particles would be below 0.5 mg/m^3^, but clearly this should be corroborated by further studies in a lower concentration range.

## Data Availability

The datasets used and/or analyzed during the current study are available from the corresponding author on reasonable request.

## Abbreviations

8-iso-PGF_2α_: 8-isoprostane
Al_2_O_3_: Aluminium oxide
CI: Confidence interval
CRP: C-reactive protein
FeNO: Fractional exhaled nitric monoxide
IL-6: Interleukin-6
IL-8: Interleukin-8
LOQ: Limit of quantification
MAD: Median absolute deviation
MMP-9: Matrix metalloproteinase
NaCl: Sodium chloride
NOEL: No observed effect level
NO_x_: Nitrogen oxides
r_S_: Spearman correlation coefficients
SAA: Serum amyloid A
TIMP-1: Tissue inhibitors of metalloproteinases
ZnO: Zinc oxide
